# Lung ultrasound: are we diagnosing too much?

**DOI:** 10.1186/s13089-023-00313-w

**Published:** 2023-03-29

**Authors:** Giovanni Volpicelli, Thomas Fraccalini, Luciano Cardinale

**Affiliations:** 1 Department of Emergency Medicine, San Luigi Gonzaga University Hospital, Turin, Italy; 2 Department of Oncology, Radiology Unit, San Luigi Gonzaga University Hospital, Turin, Italy

## Abstract

The clinical use of lung ultrasound (LUS) has made more efficient many diagnostic processes at bedside. The great power of LUS is a superior diagnostic sensitivity in many applications, when compared to chest radiography (CXR). The implementation of LUS in emergency is contributing to reveal a growing number of radio-occult pulmonary conditions. In some diseases, the superior sensitivity of LUS is a great advantage, like for pneumothorax and pulmonary edema. Diagnosing at bedside pneumothoraxes, pulmonary congestions, and COVID-19 pneumonia that are visible by LUS but undetected by CXR may be decisive for appropriate management, and even for saving lives. However, in other conditions, like bacterial pneumonia and small peripheral infarctions due to subsegmental pulmonary embolism, the high sensitivity of LUS does not always lead to advantages. Indeed, we doubt that it is always necessary to treat by antibiotics patients suspected of lower respiratory tract infection, who show radio-occult pulmonary consolidations, and to treat by anticoagulation patients with small subsegmental pulmonary embolism. The possibility that we are overtreating radio-occult conditions should be investigated with dedicated clinical trials.

## Text

Recent advances in point-of-care lung ultrasound made this technique highly impactful in the clinical practice. The advent of the modern use of lung ultrasound represents one of the most impactful and innovative novelties of the last 20 years in the field of the bedside diagnostic process in intensive care and emergency medicine [1]. The power of lung ultrasound lies in the simplicity of the technique, which may be easily learned even without being an expert ultrasound operator, and the rapid learning of the basic signs.

Lung ultrasound is mainly based on the interpretation of artifacts. Nevertheless, the diagnostic impact is solid in many applications, especially in emergency and critical conditions. The main power of lung ultrasound, demonstrated in several studies, is the excellent value of sensitivity when compared to the conventional radiologic imaging of the chest [2]. There are several applications where lung ultrasound demonstrated a higher sensitivity in comparison with chest radiography, while sharing the same high specificity. In some cases, this superior sensitivity is highly impactful because reducing the number of false-positive exams allows for an increase in the efficiency of the diagnostic process and even leads to saving lives in extreme emergency situations.

For instance, one of the applications where lung ultrasound demonstrated a great sensitivity is the bedside diagnostic process for pneumothorax. Lung ultrasound, based on the combination of three basic signs, namely lung sliding, lung pulse, and B-lines or consolidations, allows for ruling out quickly and reliably the condition of pneumothorax [3]. In this application, the sensitivity of lung ultrasound is significantly higher than bedside chest radiography [4]. Thus, using lung ultrasound in extreme emergency situations allows for diagnosing radio-occult pneumothoraxes that, in some cases, may be drained quickly; this is a procedure that sometimes may save lives or prevent deterioration in intubated patients. The advantage of using lung ultrasound in this condition is well acknowledged, and lung ultrasound not only has superior sensitivity, but it is also quicker and less costly than chest radiography.

Another application where the high sensitivity of lung ultrasound gives great advantages is the diagnosis of interstitial syndromes. For instance, in the diagnostic approach to decompensated heart failure, detection of B-lines by bedside lung ultrasound is a powerful tool to confirm the diagnosis in doubtful cases, with a far higher sensitivity than chest radiography [5]. The application of lung ultrasound can significantly improve the bedside diagnostic workup’s efficiency in emergency and help in addressing the appropriate treatment. The same can be said for the diagnosis of COVID-19 pneumonia, where lung ultrasound for B-lines was demonstrated to be far superior in sensitivity to chest radiography and allowed the correct diagnosis in a high percentage of cases with radio-occult lung involvement [6]. This latter application represented a clear advantage during the outbreak for the triage and early in-hospital allocation of patients suspected of COVID-19.

However, there are other applications where the advantages of bedside lung ultrasound, even considering the superiority to chest radiography, may be questioned. There are two main examples of this controversy.

The first example is the use of bedside lung ultrasound in the diagnosis of bacterial pneumonia in children and adults. In this application, lung ultrasound again demonstrated a specificity close to chest radiography, but a far better sensitivity when performed at bedside in critically ill patients and in emergencies [7]. Like for pneumothorax, there is a category of radio-occult conditions due to consolidations caused by lower respiratory tract infections that are visible by bedside lung ultrasound and not detected at chest radiography; this is quite commonly encountered in clinical practice [8]. Apparently, lung ultrasound represents a great advantage because of the possibility of reducing the number of false negatives at the first examination of suspected cases. However, the question arises about the need to manage this subgroup of patients with the same treating strategy that is applied for lung infections diagnosed at chest radiography. Should those consolidations visible by lung ultrasound and not by chest radiography be treated as bacterial pneumonia? In some situations, we have no doubt that antibiotics are appropriate. For instance, a multi-pathologic elderly patient with clinical signs of infection and a typical ultrasound pattern of a peripheral consolidation with dynamic air bronchogram not detected by radiology needs to be treated. Instead, for other situations we remain doubtful. For instance, in the case of a young, otherwise healthy patient with fever and cough showing a sonographic small peripheral consolidation, the antibiotic treatment may be controversial. The hesitation is felt even more in pediatric patients. The probability of radio-occult conditions in pediatric patients with signs of respiratory infection is higher than that in adults for the possibility of bronchitis and bronchiolitis, giving small peripheral atelectasis that may simulate pneumonia by ultrasound [9]. The application of a very sensitive bedside imaging tool is likely to increase the use of unnecessary antibiotics. Nowadays, the problem of rationalizing the use of antibiotics in the era characterized by a rapid and worrying increase in the phenomenon of resistance is particularly felt as a contingency.

The second example is the use of bedside lung ultrasound in the diagnostic workup of pulmonary embolism. Bedside multiorgan ultrasound of lung, heart, and deep veins can be used to improve the diagnostic workup of suspected pulmonary embolism by increasing the efficiency of the pre-test probability scoring [10]. Moreover, it is possible to use lung ultrasound for the diagnosis when an angio-CT scan is unavailable or not feasible [11]. The role of lung ultrasound is to detect peripheral infarctions representing the downstream effect of the pulmonary arterial thrombus [12]. Small infarctions are often visible by lung ultrasound and invisible by chest radiography. The higher sensitivity of ultrasound is particularly impactful in the first diagnosis of patients with mild symptoms but active acute pleuritic pain [13]. Thus, lung ultrasound implementation in emergency is bringing to an increase of mild pauci-symptomatic patients showing the typical sonographic infarction, then confirmed by multirow angio-CT as small subsegmental embolisms. The use of advanced CT devices is also reducing the number of missed diagnoses because the advent of new technologies dramatically improved the resolution of lung arteries imaging, still maintaining, or even reducing, the magnitude of patients’ irradiation [14]. Thus, thanks to the combination of lung ultrasound and modern multirow CT, sensitivity to the diagnosis of pulmonary embolism is increased. This improvement is so significant that the natural history of pulmonary embolism has changed from the disease that was reported in the past as a very dangerous, often deadly condition. We now diagnose many more cases of mild pulmonary embolisms that are not evolutive and do not represent a danger to the patient’s life. Again, like for pneumonia, a question arises: do we need anticoagulation in all these cases, like still recommended in the societal guidelines? Anticoagulants expose the patients to collateral risks that should be weighed against the probability of adverse thromboembolic events [14]. However, prognosis of mild subsegmental embolisms cannot be compared to that of severe forms with hemodynamic impairment. For instance, a young woman taking contraceptives who presents with acute pleuritic pain and with the absence of respiratory signs, revealing at lung ultrasound a typical small peripheral wedge-based consolidation corresponding to the painful area, will be sent to angio-CT scan in the strong suspicion of embolism. Quite often in these patients, CT will confirm the diagnosis of a small subsegmental embolism. The use of contraceptives represents the main etiology, but we do not know the evolutionary potential of the thromboembolic disease. By extending the multiorgan ultrasound evaluation to the heart and deep veins, it is possible to ascertain signs of right ventricle impairment and presence of thrombi still active in the deep veins. When these two examinations are negative, the probability of relapse and hemodynamic consequences is extremely low. Does a young woman, who will stop contraceptives for the rest of her life, still need to be anticoagulated? How convenient is prescribing anticoagulation for a disease that will have no more causes for relapse and have a very low probability of further evolution? Until now, there is no evidence-based answer to these questions. Besides the unnecessary risk of anticoagulation, there is also the problem of adverse effects using contrast medium and irradiation by angio-CT. There is a growing number of patients with mild forms of embolism who perform angio-CT for confirmation, which increases the occurrence of side effects, like allergic shock and renal failure from contrast medium, and cancer from irradiation [14].

## Conclusion

In recent years much progress has been made that affirmed the diagnostic role of bedside point-of-care lung ultrasound. In many applications, lung ultrasound demonstrated higher sensitivity than chest radiography, revealing many radio-occult conditions that may otherwise remain undiagnosed. In some of these applications, the higher sensitivity of ultrasound imaging represents an unquestionable advantage, like for pneumothorax and pulmonary edema. In some others, the diagnosis of radio-occult pulmonary conditions poses questions about the appropriateness of common conventional treatments. In situation where a therapeutic regimen is burdened by the possibility of side effects, like the use of antibiotics for radio-occult pneumonia or anticoagulants for peripheral subsegmental embolisms, this unanswered question demands urgent response by dedicated clinical research.

## Data Availability

Not applicable.
